# Prior Extracorporeal Membrane Oxygenation (ECMO) Experience and Performance in High-Fidelity Simulation Scenarios

**DOI:** 10.7759/cureus.29301

**Published:** 2022-09-18

**Authors:** Emma Prichard, Amanda M Staudt, Tricia Garcia-Choudary, Thornton Mu

**Affiliations:** 1 Pediatrics, Brooke Army Medical Center, Fort Sam Houston, USA; 2 Clinical Research Support, U.S. Army Institute of Surgical Research, Fort Sam Houston, USA; 3 Neonatal-Perinatal Medicine, Brooke Army Medical Center, Fort Sam Houston, USA

**Keywords:** just-in-time training, simulation in medical education, skills and simulation training, ecmo complication, ecmo, critical care

## Abstract

Background

Extracorporeal Membrane Oxygenation (ECMO) provides a heart-lung bypass for patients with life-threatening cardiorespiratory failure. It is a classic low-volume, a high-risk procedure that requires specialized training to develop and maintain competence. Therefore, our ability to train efficiently and effectively is essential. The purpose of this study is to determine if specific participant training or experience leads to better performance in emergency ECMO scenarios during high-fidelity simulation training.

Methods

Fifty-one physicians, nurses, and respiratory/medical technicians participated in a study comparing an animal model vs. simulation-based ECMO education. All completed a multiple-choice questionnaire about prior ECMO experience and other demographics, as well as a four-hour pre-lab didactic session. They completed individual ECMO scenarios with both modalities during two sessions, and task completion times (minutes) and scores (percentage) were measured using a validated ECMO skills assessment tool. The scores of the 19 participants who completed the simulation-based scenarios during their first session were further analyzed in the context of their self-reported ECMO experience, and participants were divided into a novice group and an experienced group. Statistical testing included the Mann-Whitney U test (times) and Fisher’s exact test (scores).

Results

Data from the 19 participants who completed the simulation-based ECMO training on the first session showed no statistically significant differences in the task completion time or scores among those in the novice group vs. the experienced group in the years of ECMO experience category (28 vs. 34 minutes; p=0.66 and 61% vs. 62%; p=0.54), a number of cannulations category (30 vs. 25 minutes; p=0.11 and 59% vs. 62%; p=0.82) or the number of ECMO patients cared for category (28 vs. 34 minutes; p=0.30 and 57% vs. 62%; p=0.54). Findings were similar for both the lecture-based training and simulation-based training categories, respectively (33 vs. 28 minutes; p=0.71 and 62% vs. 60%; p=0.91 and 34 vs. 28 minutes; p=0.74 and 63% vs. 58%; p=0.12).

Conclusion

Among this small subset of participants, we observed no statistically significant differences in performance based on participant experience during simulation-based ECMO scenarios. The didactic/review sessions preceding the training may have contributed to an effective form of training for participants with no prior ECMO experience. Due to the small sample size of this study, further studies are needed to better elucidate what factors lead to better performance in emergency ECMO scenarios.

## Introduction

Extracorporeal Membrane Oxygenation (ECMO) is a low-volume, high-risk procedure that provides a heart-lung bypass for patients with life-threatening cardiorespiratory failure. As advanced pulmonary and cardiovascular support measures evolve, ECMO is reserved as a last resort therapy, meaning current providers and trainees have limited opportunities to learn and practice the unique life-saving skills necessary to manage these complex patients and avoid complications that adversely affect survival rates [1]. Therefore, our ability to train clinicians efficiently and effectively is essential. Historically, training has utilized animal models in combination with ECMO didactic education to facilitate both the acquisition and sustainment of the required skills. More recently, simulation models have demonstrated increased sophistication and promise [2-3]. Mu et al. showed in 2020 that both animal and simulation models demonstrate benefits in the development of and/or maintaining skill competence, with no significant differences in participant performance between the two modalities. High-fidelity simulation training may be an effective and less costly option for ECMO training compared with animal models [4]. The purpose of this study is to determine if specific participant training or experience leads to better performance in emergency ECMO scenarios during high-fidelity simulation training. This article was previously presented as a meeting abstract at the 2021 American Academy of Pediatrics National Conference on October 8, 2021.

## Materials and methods

This study was a secondary analysis of a data set from a previously published study [4]. Briefly, the original study used a block randomized crossover training protocol comparing an animal training model versus a simulation training model. Participants underwent sequential randomization to either Track A (animal lab first; simulation lab second) or Track B (simulation lab first; animal lab second). They then completed several ECMO scenarios on each training modality designed to simulate common emergent situations unique to the ECMO circuit. This study analyzed a subset of data from 19 participants who completed the simulation-based lab training experience first (Track B). The data from the animal model were excluded to reduce the chance of variability between the two models, and only the first attempt was analyzed to avoid any inherent performance improvement between the first and second training iterations.

Participants initially completed a multiple-choice questionnaire delineating their clinical discipline, duration of ECMO experience, ECMO cannulation experience, and prior ECMO training (lecture-based or simulation-based). They then participated in a four-hour pre-lab didactic session consisting of multiple video webinars covering ECMO cannulation, emergency procedures, and clinical checklists, as well as a demonstration of appropriate cannulation techniques and orientation to the training environment. Before completing the scored scenarios, they also had one opportunity to review the cannulation and orientation videos and to practice pump failure maneuvers.

The following four scenarios were included (1) poor venous return, (2) gas failure, (3) pump failure, and (4) arterial air. Each scenario required the participant to first identify the problem and then complete the appropriate steps, tailored for each of the four disciplines, to fix or troubleshoot the problem. Performance was then measured based on four different variables (1) task time: the total time (minutes) it took to complete all the required tasks in the scenario (2) critical task time: the time it took to complete tasks that were deemed as critical in identifying and successfully completing the scenario (3) task completion: the percent of required tasks that were completed during the scenario (4) critical task completion: the percent of critical tasks that were completed during the scenario. This scoring system was developed using an ECMO skills assessment tool created for the original study by the research team and validated by three ECMO subject matter experts not involved in the study using Kane’s validity framework [5-6]. Performance was reported as a percentage based on the number of tasks completed (total and critical) as well as the time needed to complete the tasks (total and critical). Higher percentages and quicker times were considered better performance in the scenario. The scoring was completed by facilitators who completed training on the scenarios and scoring system. They were present when participants completed the scenarios. See Appendix A for details on the simulation trainer used, scenarios, and assessment tool.

The 19 participants were stratified by years of experience, number of ECMO patients cared for, number of cannulations, number of prior lecture-based ECMO training, and number of prior ECMO simulation-based training based on questionnaire results. Within these categories, they were then divided into three groups: novice, intermediate and advanced. The Novice group included those who identified as having zero years of experience, no ECMO patients, no cannulations, and no prior lecture-based or simulation-based training. The Intermediate group reported one to five years of experience, patients cared for, cannulations or prior lecture-based or simulation-based training, and the Advanced group had greater than five. Due to the few numbers of participants in the Advanced group, we chose to redistribute participants and compare a Novice group (no prior years, patients, cannulations, training) and an Experienced group (those with one or more years, patients, cannulations, or training).

Statistical analysis

In the un-stratified analysis, we reported counts (percentages) and medians (quartile 1 (Q1) and quartile 3 (Q3)) for categorical and continuous variables, respectively. Median (Q1-Q3) task completion times and number (percent) of tasks completed for both total and critical tasks were stratified by self-reported ECMO experience (years of experience, number of patients, number of cannulations, number of lecture-based training, and number of simulation-based training). Statistical testing for continuous variables stratified by three or more groups used the Kruskal-Wallis exact test, while variables stratified by two groups used the two-sample Wilcoxon exact test. 

## Results

Nineteen participants completed the four simulation scenarios and were included in the analysis. The group was made up of two attending physicians (10.5%), seven residents/fellows (36.8%), four specialists (21.1%), and six technicians (31.6%). Participant demographics and self-reported ECMO experience are reported in Table 1. In each category, the majority of participants reported having no ECMO experience.

**Table 1:** Demographics of Study Participants RT: Respiratory therapist; MT: Medical technician; ICURN: Intensive care unit registered nurse; ECMO: Extracorporeal membrane oxygenation

Demographics of study participants	n
Attending	2
Resident	7
Specialist	4
RT/MT/ICURN	6
Years of ECMO experience	
Novice	11
Intermediate	7
Advanced	1
Number of previous ECMO patients	
Novice	7
Intermediate	6
Advanced	6
Number of previous ECMO cannulations	
Novice	14
Intermediate	4
Advanced	1
Number of previous ECMO lecture-based cannulations	
Novice	12
Intermediate	6
Advanced	1
Number of previous ECMO mannequin-based simulation training sessions	
Novice	15
Intermediate	3
Advanced	1

There were no differences in the task completion time or percent among the novice group vs. experienced group in the years of ECMO experience category (28 vs. 34 minutes; p=0.66 and 61% vs. 62%; p=0.54). There was also no difference in those who had never cared for an ECMO patient vs. those who had cared for at least one (28 vs. 34 minutes; p=0.30 and 57% vs. 62%; p=0.54) or those with no vs. ≥ one cannulation (30 vs. 25 minutes; p=0.11 and 59% vs. 62%; p=0.82). Individuals in the novice group for lecture-based training did not perform significantly better than those in the experienced group (33 vs. 28 minutes; p=0.71 and 62% vs. 60%; p=0.91). This was also true for those simulation-based training category (34 vs. 28 minutes; p=0.74 and 63% vs. 58%; p=0.12). Summarized results are depicted in Table 2.

**Table 2:** Task Completion Time or Percent in Novice Group and Experienced Group *Technical difficulties impacted data collection resulting in variable ‘n’ values reflected in the table ECMO: Extracorporeal membrane oxygenation; Q1: Quartile 1; Q3: Quartile 3

Task Completion Time or Percent in Novice Group and Experienced Group*					
Years of ECMO Experience	Novice		Experienced		
	n	Median (Q1-Q3)	n	Median (Q1-Q3)	p value
Task time, minutes	11	28.2 (26.8-32.7)	8	34.3 (25.0-47.1)	0.66
Critical task time, minutes	6	18.0 (17.7-19.9)	5	22.4 (16.0-32.1)	0.54
Task completion difference, %	11	0.61 (0.45-0.64)	8	0.62 (0.56-0.68)	0.54
Critical task completion difference, %	11	0.98 (0.90-1.0)	8	0.96 (0.94-0.97)	0.55
Number of ECMO Patients	Novice		Experienced		
	n	Median (Q1-Q3)	n	Median (Q1-Q3)	p value
Task time, minutes	7	27.9 (26.8-31.0)	12	34.3 (25.7-45.8)	0.3
Critical task time, minutes	2	18.0 (17.7-19.9)	9	19.9 (16-29.1)	0.6
Task completion difference, %	7	0.57 (0.49-0.63)	12	0.62 (0.56-0.66)	0.5
Critical task completion difference, %	7	0.94 (0.81-1.0)	12	0.96 (0.94-0.98)	0.5
Number of ECMO Cannulations	Novice		Experienced		
	n	Median (Q1-Q3)	n	Median (Q1-Q3)	p value
Task time, minutes	14	30 (26.9-44)	5	25.2 (24.8-32.7)	0.1
Critical task time, minutes	8	21.1 (17.9-30.6)	3	16 (15.7-17.8)	0.1
Task completion difference, %	14	0.59 (0.54-0.64)	5	0.62 (0.62-0.63)	0.8
Critical task completion difference, %	14	0.96 (0.92-1.0)	5	0.96 (0.94-0.98)	0.7
Number of ECMO Lecture-based Certifications	Novice		Experienced		
	n	Median (Q1-Q3)	n	Median (Q1-Q3)	p value
Task time, minutes	12	28.1 (26.4-34.7)	7	33.0 (25.2-44.0)	0.71
Critical task time, minutes	7	17.8 (15.7-29.1)	4	20.2 (17.1-29.3)	0.53
Task completion difference, %	12	0.6 (0.54-0.64)	7	0.62 (0.54-0.64)	0.96
Critical task completion difference, %	12	0.97 (0.92-1.0)	7	0.96 (0.92-0.98)	0.44
Number of Prior ECMO-based Simulations	Novice		Experienced		
	n	Median (Q1-Q3)	n	Median (Q1-Q3)	p value
Task time, minutes	15	28.1 (26.1-36.8)	4	34.3 (28.4-39.8)	0.7
Critical task time, minutes	9	18.1 (17.7-29.1)	2	19.2 (16.0-22.4)	0.9
Task completion difference, %	15	0.58 (0.54-0.64)	4	0.63 (0.62-0.72)	0.1
Critical task completion difference, %	15	0.94 (0.9-1.0)	4	0.97 (0.96-0.98)	0.5

## Discussion

In medical as well as other highly specialized fields, one assumes that more experience and training result in better performance outcomes. Studies have demonstrated improved ECMO knowledge and/or performance following participation in a training course utilizing simulation models [7-13]. We sought to understand if prior ECMO experiences and/or training would lead to better performance. Our analyses showed that performance in four emergency ECMO simulation scenarios was not statistically significantly different among participants with varied training and experience.

We speculate the didactic and review session preceding training enabled novice ECMO providers to perform at a level that was not significantly different from those in the experienced group, similar to what we have seen in prior research on “just in time” training with other high-risk, low volume procedures [14-15]. Maddry et al. were able to demonstrate similar benefits of a “just in time” style training when applied to ECMO. Their study included 34 physicians and nurses with no prior formal ECMO training who were able to successfully prime and initiate ECMO circuits on a live-tissue simulation following the completion of an abbreviated eight-hour ECMO course [16]. Emergency medicine physicians and nurses with no prior ECMO experience were able to rapidly and safely initiate ECMO both immediately and three months after a two-day training course in a study published in Resuscitation in 2019 [17]. These studies suggest that this type of abbreviated or “just in time” training could be an effective way to achieve proficiency in these low-volume, high-risk procedures. Our study adds to the growing evidence in favor of this style of training for clinicians and trainees in the medical field.

Our study has several strengths. We included a multidisciplinary cohort to participate in this project that utilized a high-fidelity simulation training platform. We developed and incorporated four complex individual skill scenarios with built-in learner feedback as part of a comprehensive training curriculum. We collected validity evidence for our ECMO skills assessment tool following Kane’s validity framework for educational assessments [5-6]. We also identified several limitations to our study. First, most of the study participants were novices regarding ECMO experience, making it difficult to create a truly experienced group with a sample size large enough for meaningful comparison. Therefore, the groups were divided into those with no experience and those with at least one year of experience. This limits the generalizability of our findings and could explain why no significant differences were uncovered. Our decision to analyze only participant performance from the simulation lab (Track B) also created limitations to our study as it did not allow us enough participants to achieve the power necessary to reach statistical significance. 

## Conclusions

Among this small subset of participants, performance was similar in emergent ECMO simulation scenarios, regardless of prior training or experience. Didactic and review sessions before training may be an effective educational strategy for novice participants. Further studies are needed to better elucidate what types of training/experience lead to better performance in emergency ECMO scenarios.

## Appendices

Appendix A

Excerpts From Original Study Added For Clarification [4]

Simulation lab: The ECMO simulator system consisted of a high-fidelity synthetic tissue cannulation task trainer (SynDaver Labs, Tampa, Florida), connected to a water-tight closed reservoir pump, embedded into a low-fidelity newborn infant manikin (Laerdal Medical Corporation, Wappingers Falls, New York, USA) system. This prototype was optimized to advance the realism of the simulation model for this study and consisted of life-like skin and subcutaneous fat overlaying the sternocleidomastoid muscle, which was retracted to reveal a medially placed carotid sheath containing the internal jugular vein, the vagus nerve, and the common carotid artery. The internal jugular vein (13Fr vessel caliber) and common carotid artery (8Fr vessel caliber) were isolated, incised, and cannulated with an ECMO cannula. The major vessels are ‘dead-ended’ peripherally into a water-tight closed reservoir system. Once the cannula was in place, they were connected to a similar ECMO circuit described above except primed with a simulated blood product solution. Simulation operators controlled pressure transducers (Fogg System Company, Aurora, Colorado, USA) and adjusted patient display software (Laerdal Medical Corporation) to display ‘real-time’ patient vital signs and circuit pressure measurements.

ECMO Training Scenarios 

Participants completed four individual scenarios during each lab, detailed below: 

1. Poor venous return: this scenario required participants to recognize bleeding at the cannulation site, contributing to poor venous return. Pressure transducers were used to increase the negative venous pressures that shut down the ECMO pump when pressure alarm limits were exceeded. Participants were required to recognize increasingly negative venous pressure and to provide appropriate intervention.

 2. Gas failure: this scenario required participants to troubleshoot the patient’s oxygen desaturations to identify a disconnected gas source and reconnect it.

 3. Pump failure: a simulated power outage and subsequent surge disabled the ECMO pump and required the participant to initiate manual pumping of the circuit with the hand crank and place the patient back on ECMO with a new ECMO pump.

 4. Arterial air: this scenario required the participant to recognize air on the arterial side of the ECMO, take the patient off ECMO and effectively remove all air from the circuit. Participants were also required to describe how to correct an air embolism in the venous side of the ECMO circuit.

Scoring

Objective performance was based on a total weighted performance score derived from an ECMO skills assessment tool created for this study by the research team, and content validity was established via review by three ECMO subject matter experts not involved in the study. The tool was constructed in a weighted Go/No Go checklist format for each step, with a preassigned priority value (basic or critical) for weighted scoring. Additionally, these performance skills were tailored for each of the four disciplines. Critical performance measures were also timed for task completion. All facilitators and instructors completed a simulation education course, received extensive training on both the simulator and the animal model, and watched a sample videotaped performance of optimal execution of the six scenarios enacted by an ECMO clinician not involved in the study. Facilitators and instructors were also responsible for prestaging each scenario. To achieve appropriate inter-rater reliability, facilitators scored the sample videotape performance and were required to achieve 80% concordance before evaluating any study participants. To eliminate variability in the degree of assistance provided to each participant, instructors followed a precise script for each scenario, which provided the simulated patient’s clinical history and allowable responses to clinical queries (not evident on a patient exam or disabled on the monitoring equipment) and permitted prompting to elicit knowledge check items not spontaneously demonstrated. Instructors debriefed each scenario ranging from two to five min. to provide individually tailored educational feedback encompassing teamwork, clinical as well as technical skills. During the study labs, two facilitators evaluated the participants. Following each scenario, facilitators reviewed their scores to ensure concordance and adjudicate any discrepant scoring. Female and male facilitators and instructors were critical care attending physicians and perfusion-trained ICU nurses. Video recordings of the participants were not used.
